# Progress of Breast Cancer basic research in China

**DOI:** 10.7150/ijbs.60631

**Published:** 2021-05-11

**Authors:** Xuerong Wang, Chao Wang, Jiaheng Guan, Baoan Chen, Lin Xu, Ceshi Chen

**Affiliations:** 1 Department of Pharmacology, Nanjing Medical University, Nanjing, Jiangsu 210029, China.; 2 Department of Thoracic Surgery, Jiangsu Cancer Hospital, Nanjing Medical University Affiliated Cancer Hospital, Nanjing, Jiangsu 210009, China.; 3 Department of Hematology and Oncology, Zhongda Hospital, School of Medicine, Southeast University, Nanjing, Jiangsu 210009, China.; 4 Key Laboratory of Animal Models and Human Disease Mechanisms of the Chinese Academy of Sciences and Yunnan Province, Kunming Institute of Zoology, Chinese Academy of Sciences, Kunming, Yunnan 650223, China.

**Keywords:** breast cancer, cancer stem cells, tumor microenvironment, metastasis, drug resistance

## Abstract

Breast cancer is the most commonly diagnosed and the most lethal cancer in females both in China and worldwide. Currently, the origin of cancer stem cells, the heterogeneity of cancer cells, the mechanism of cancer metastasis and drug resistance are the most important issues that need to be addressed. Chinese investigators have recently made new discoveries in basic breast cancer researches, especially regarding cancer stem cells, cancer metabolism, and microenvironments. These efforts have led to a deeper understanding of drug resistance and metastasis and have also indicated new biomarkers and therapeutic targets. These findings emphasized the importance of the cancer stem cells for targeted therapy. In this review, we summarized the latest important findings in this field in China.

## Introduction

Breast cancer is the most common malignant disease in women worldwide. According to the data released in 2019, breast cancer accounted for 30% of newly diagnosed malignant tumors in females, and led to 15% of female deaths from cancer 1. The incidence and mortality of breast cancer are still rising, both in developing and developed countries 2. In China, breast cancer morbidity and mortality rank first among malignant diseases in females. Death rate reached about 6.9% among all female malignant tumors 3. The lack of early-stage screening and detection methods and cost-effective therapies makes breast cancer as one of the most severe disease burden globally 4. According to the international consensus guideline, the current treatment for breast cancer mainly included chemotherapy, radiotherapy, targeted therapy, immunity therapy, and endocrine therapy before and after surgery 5. Nowadays, therapies targeted to cancer stem cells have been a hot spot in breast cancer treatment, which is a supplement to the traditional chemotherapeutic drugs that are unable to eradicate tumor dissemination and metastasis 6, 7. Great advances in cancer cell-of-origin, somatic mutation, epigenetic alteration, and tumor microenvironment were revealed in recent studies, both basic and clinical, leading to a better understanding of the mechanism, the pathogenesis, the diagnosis, and the treatment of breast cancer. Particularly, Chinese investigators have made great achievements in cancer stem cells, microenvironment, metastasis, drug resistance, tumor biomarkers, and new targets, as well as drugs for breast cancer therapy. In this review, we summarized the important findings on breast cancer basic research in China from 2019 to 2020.

## Cancer stem cells (CSCs)

**Figure 1:**
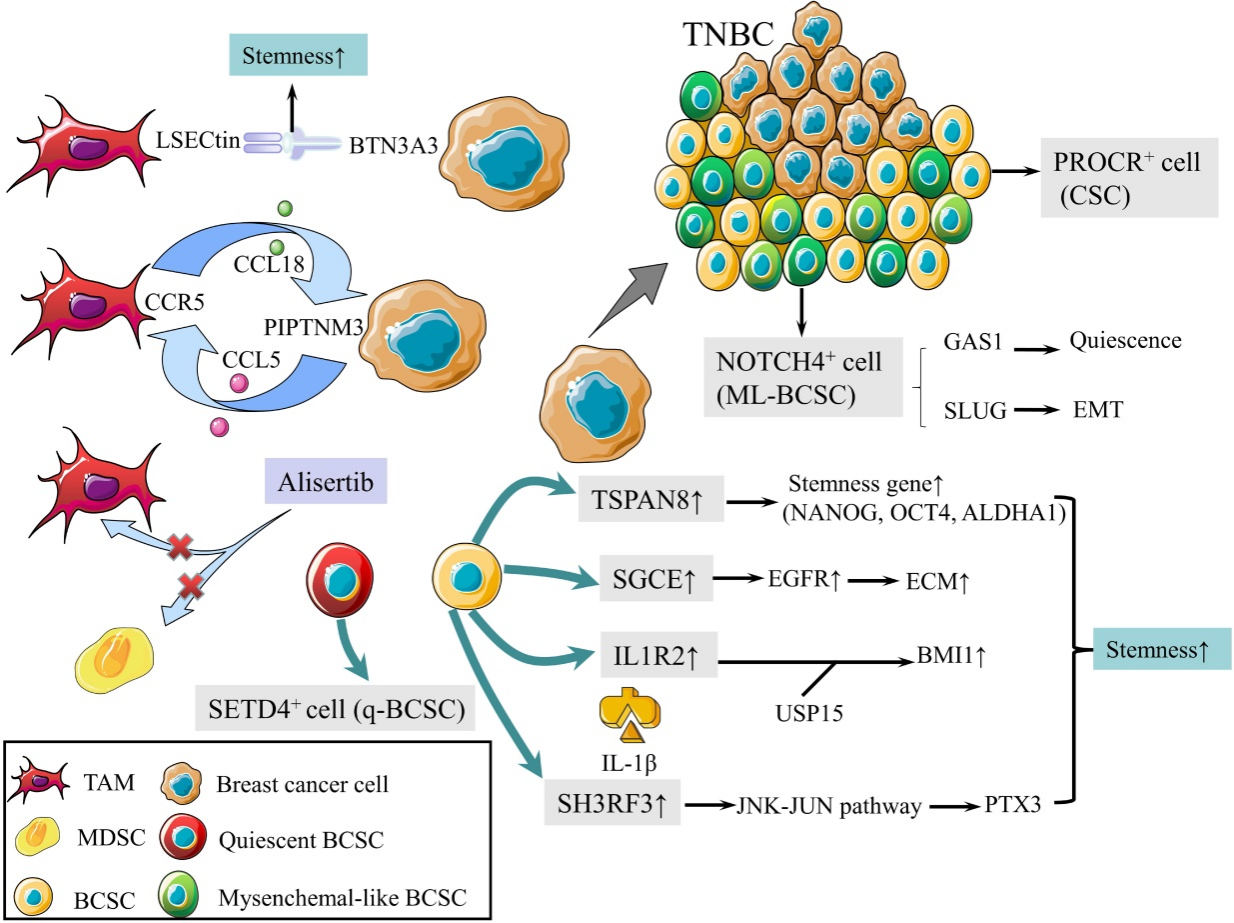
** Schematic diagram of BCSCs and TME.** (1) PROCR is a marker of BCSCs for stratifying TNBC into subgroups. (2) NOTCH4 is also a marker for BCSCs, driving ML-BCBCs into a quiescent state via GAS1 and inducing EMT via SLUG. (3) TAMs with elevated LSECtin expression interacts with BTN3A3, promoting the stemness of breast cancer cells. (4) The following pathways increase cell stemness. IL1R2 interacts with USP15 to induce deubiquitination and stabilization of BMI1. SGCE stabilizes EGFR. TSPAN-8 enhances stemness genes, including NANOG, OCT4, and ALDHA1. SH3RF3 activates PTX3 via the JNK-JUN pathway. (5) BCSCs are divided into e-BCSCs and q-BCSCs. SETD4 is important in the maintenance of qBCSCs. (6) A positive feedback loop between CCL5-CCR5 and CCL18-PIPTNM3 is shown. TAMs secret CCL18, binding to PIPTNM3 on breast cancer cells. Then breast cancer cells secret CCL5, inducing macrophages to a TAM-like phenotype CCR5. (7) Alisertib eliminates tumor-promoting MDSCs and TAMs to reshape the immune microenvironment.

CSCs are defined as a group of undifferentiated cells that possess properties of self-renewal and pluripotent differentiation. Although breast cancer stem cells (BCSCs) make up only a small subpopulation within tumors, they are responsible for tumor initiation, recurrence, metastasis, and therapy resistance 8. Previous studies revealed a panel of biomarkers for BCSCs identification, among which CD44^high^, CD24^low^ and aldehyde dehydrogenase 1 (ALDH1)^+^ were the most commonly used biomarkers 9. Triple-negative breast cancer (TNBC) contains more BCSCs than other subtypes, which is associated with worse outcomes 10, 11. In 2019, protein C receptor (PROCR) was identified as a novel marker for BCSCs, which could stratify TNBC into clinically relevant subgroups and be a therapeutic target (Fig. 1) 12. NOTCH4 receptor was not only implicated in the regulation of BCSCs in TNBC, but also regarded as a better marker for BCSCs than CD24^-^CD44^+^ in 2020 (Fig. 1). Mechanistically, NOTCH4 drives mesenchymal-like BCSCs into a quiescent state and induces epithelial-mesenchymal transition (EMT) via upregulating GAS1 and SLUG, respectively. Zhou et al. also demonstrated the importance of NOTCH4-SLUG-GAS1 circuit in maintaining mesenchymal-like BCSCs 13.

Tumor microenvironment (TME) plays an essential role in regulating the activity of BCSCs 14. Tumor-associated macrophages (TAMs) with elevated LSECtin expression interacts with its receptor BTN3A3, expressed in cancer cells, to promote the stemness of breast cancer cells in a cell-cell contact dependent manner (Fig. 1) 15. In addition, the Jag1 expression in endothelial cells activates Notch 1 to upregulate Zeb1 expression and to increase vascular endothelial growth factor A (VEGFA) production. In turn, VEGFA induces Jag1 expression in endothelial cells. Thus, the positive feedback loop in the tumor perivascular niche promotes the stemness of breast cancer cells 16.

There are other pathways to enhance the stemness of BCSC. Activated interleukin-1 receptor type 2 (IL1R2) interacts with deubiquitinase USP15 (ubiquitin-specific protease 15) to induce deubiquitination and stabilization of BMI1, which facilitates the self-renewal of BCSCs (Fig. 1) 17. BCSCs were also promoted by SGCE via an interaction with c-Cbl and the inhibition of c-Cbl-mediated epidermal growth factor receptor (EGFR) lysosomal degradation (Fig. 1) 18. The elevated stress sensor in response to hypoxia, Tribble 3 (TRIB3) promoted BCSCs through AKT1-FOXO1 (forkhead box O1)-SOX2 (sry-related high mobility box 2) axis 19. Phospholipid scramblase 1 (PLSCR1) enhanced stem cell-like properties through upregulating signal transducer as well as activator of transcription 1 (STAT1) expression in basal-like breast cancer (BLBC) 20. Additionally, loss of SIRT4 promoted self-renewal and expansion of BCSCs through suppressing glutamine metabolism in mitochondrial and SIRT1-mediated BRCA1 transcription in nucleus, which provided a novel cross-talk between mitochondrial and nuclear sirtuins 21.

Signaling pathways activated in BCSCs mainly include Wnt, Notch, and Hedgedog (Hh) 22. The Hh signaling pathway is activated by the binding of Hh ligands, such as Sonic Hedgehog (SHH), and their cognate receptors, such as Ptch1. Tetraspanin-8 (TSPAN-8) recruits the ATXN3 deubiquitinating enzyme to reduce the ubiquitination of PTCH1 and to inhibit the degradation of the SHH/PTCH1 complex (Fig. 1). Therefore, TSPAN8 enhances the Hh pathway and breast cancer cell stemness 23. In addition, SH3 domain containing ring finger 3 (SH3RF3) promotes BCSCs by activating the JNK (c-Jun N-terminal kinase)-JUN pathway, as well as the expression of pentraxin 3 (PTX3) (Fig. 1) 24.

BCSCs are divided into two categories according to the cell cycle rate: energetic BCSCs (e-BCSCs) 25 and quiescent BCSCs (q-BCSCs) 26. Q-BCSCs play critical roles in resistance to chemoradiotherapy and disease relapse. SET domain-containing protein 4 (SETD4) is important in the maintenance of qBCSCs (Fig. 1). SETD4 facilitates heterochromatin formation via H4K20me3 (trimethylation of lysine 20 of histone 4) catalysis on certain promoter regions which leads to the silencing of qBCSCs-suppressing genes. Notably, SETD4-defined qBCSCs maintain quiescent state by asymmetric division, producing a small qBCSC and a big active daughter cell; the latter generates normal cancer cells 27. These findings on stem cell status expanded our knowledge on the epigenetic determinants of q-BCSCs and provided new therapeutic targets for drug-resistant q-BCSCs.

## Metabolism

Altered metabolism is an emerging hallmark of cancer. Unlike normal cells, cancer cells prefer aerobic glycolysis, which is accompanied by increased lactate production, also known as the Warburg effect 28. Based on the law of conservation of electrons in chemical reactions, Li et al. built up an electron balance model to outline metabolic plasticity under hypoxia. According to the model, both proline biosynthesis and lipogenesis can act as alternative electron acceptor. Blocking them synergistically suppresses tumor growth 29. Moreover, tumor associated macrophages (TAMs) enhance aerobic glycolysis and induce apoptosis resistance of breast cancer cells via extracellular vesicle (EV) transmission of HIF-1α-stabilizing long noncoding RNA (HISLA). Reciprocally, lactate released from glycolytic cancer cells upregulates HISLA in TAMs, constituting a feed-forward loop between TAMs and cancer cells 30.

Psychological factors induced metabolic alterations was revealed to cancer progression. Lactate dehydrogenase A (LDHA) executes the final step of the Warburg effect by converting pyruvate to lactate. Breast cancer stem-like properties can be promoted by chronic stress-induced epinephrine via LDHA-dependent metabolic rewiring. Interestingly, vitamin C may reverse the chronic stress-induced cancer stem-like phenotype 31.

## Tumor microenvironment (TME)

The TME of breast cancer is composed of multiple stromal cells, soluble factors, and physical properties, including intratumor and metastatic microenvironment representing local and distant lesions, respectively 32.

In malignant phyllodes tumors (PT), TAMs promote malignant progression by secreting large amount of CCL18, which then binds to its receptor PIPTNM3 on myofibroblasts 33. Reciprocally, malignant PT recruits and induces macrophages to a TAM-like phenotype by secreting CCL5. Thus, a positive feedback loop between CCL5-CCR5 and CCL18-PIPTNM3 is constituted, which represents the communication of myofibroblasts with TAMs and plays a central role in tumorigenesis of PT (Fig. 1) 34.

Tumor development is accompanied by the occurrence and persistence of immunosuppressive microenvironment. Myeloid-derived suppressor cells (MDSCs) and TAMs interact with CD4^+^ and CD8^+^ T lymphocytes and subsequently attenuate the anti-tumor immunity response, which is one of the factors that most responsible for immune evasion. Alisertib, a selective Aurora A kinase inhibitor, could reshape the immune microenvironment through selectively eliminating tumor-promoting myeloid cells, including MDSCs and TAMs, as well as restoring the anti-tumor immunity of T lymphocytes (Fig. 1). Intriguingly, combining alisertib with anti-programmed cell death-ligand 1 (PD-L1) therapy showed a synergistic efficacy in the treatment of advanced breast cancer 35.

According to the “seed and soil” theory, premetastatic niches in destination organs are key driving force for tumor dissemination and colonization 36. Additionally, primary tumor is capable of inducing B cell accumulation in draining lymph nodes. These tumor-educated B cells produce pathogenic IgG that targets the tumor membrane antigen HSPA4 (heat shock protein family A member 4) to activate the HSPA4-binding protein ITGB5 (integrin β5) and the downstream Src/NF-κB pathway. These findings illustrated the role of B cells in promoting the establishment of premetastatic niches in draining lymph nodes and accelerating breast cancer lymph node metastasis 37.

## Tumor metastasis

**Figure 2:**
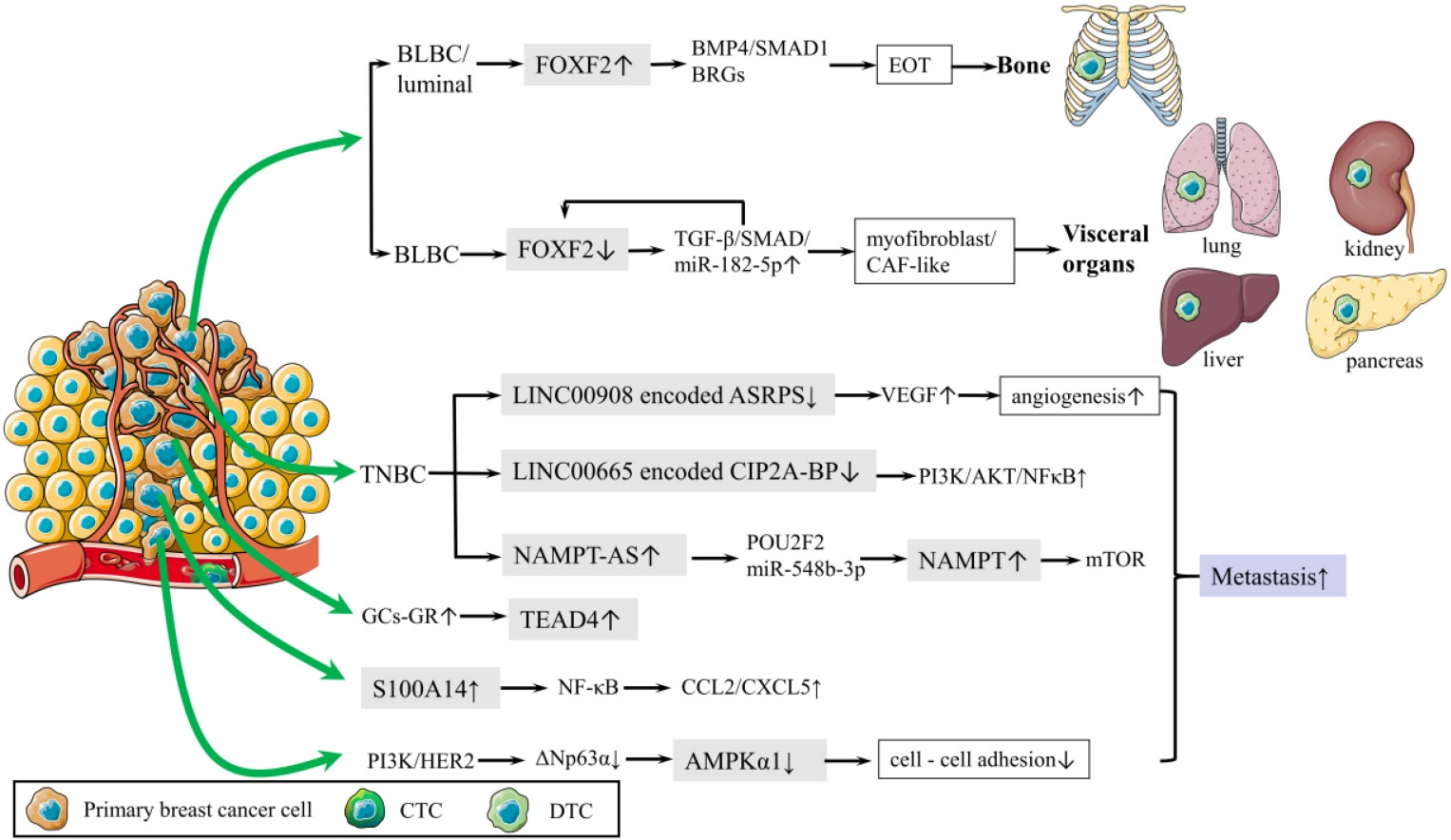
** Schematic diagram of metastasis.** (1) Mechanism of bone metastasis is shown.FOXF2 overexpressed by BLBC/luminal regulates EOT via BMP4/SMAD1 pathway and BRGs, leading to bone metastasis. (2) Mechanism of visceral metastasis is shown. Deficiency of FOXF2 in BLBC transdifferentiates to myofibroblast/cancer associated fibroblast (CAF)-like phenotype and metastasizes to visceral organs via TGF-β/SMAD/miR-182-5p, which in turn repress FOXF2. (3) PI3K and HER2 activation downregulates AMPKα1 expression via ΔNp63α, reducing cell-cell adhesion. (4) S100A14 promotes cancer metastasis by upregulating the secretion of CCL2 and CXCL5 via NF-κB pathway. (5) Mechanisms of lncRNA in regulating TNBC metastasis are shown below. Oncogenic lncRNA, NAMPT-AS activates NAMPT via POU2F2 miR-548b-3p.The reduction of LINC00665 encoded CIP2A-BP increases PI3K/AKT/NFκB to reduce lung metastasis. LINC00908 encodes ASRPS inhibits angiogenesis via VEGF.

Distant metastasis is the main cause of breast cancer related death. A recent study deepened our understanding of EMT in metastasis by revealing that breast cancer cells were capable of recapitulating various epithelial and mesenchymal phenotypes. Epithelial-type circulating tumor cells (CTCs) and disseminated tumor cells (DTCs) with a restricted mesenchymal transition show the most metastatic traits, whereas mesenchymal-type CTCs and DTCs display limited metastatic ability 34. Bone is the most common site of distant metastasis. To further explain the mechanisms underlying the predilection of bone metastasis has always been a focus of breast cancer research. It was proved that Forkhead box F2 (FOXF2) regulated the epithelium-to-osteomimicry transition (EOT) via pleiotropic transactivation of the BMP4/SMAD1 signaling pathway and bone-related genes (BRGs), thus giving the metastatic tendency to bone (Fig. 2) 38. The same group also revealed that FOXF2 deficiency BLBC tended to transdifferentiate to a myofibroblast/cancer associated fibroblast (CAF)-like phenotype and metastasized to visceral organs. The underlying mechanism involves the reciprocal repression loop between FOXF2 and transforming growth factor-β (TGF-β) (Fig. 2) 39. These groundbreaking studies provided theoretical basis for preventing breast cancer metastasis.

Metastasis-initiating cells (MIC) are a tiny population of cells, estimated less than 0.02% of disseminated tumor cells, and are responsible for forming secondary tumors. Yang et al found platelet-derived growth factor receptor (PDGFR) inhibition blocked AKT activation but not serum and glucocorticoid induced kinase 1 (SGK1) signaling in MIC, which resulted in suppressing lung metastasis of breast cancer; however, primary tumor burden was not affected. Co-targeting PDGFR and SGK1 showed synergistic anti-cancer effects and led to further inhibition of pulmonary metastases and primary breast tumors 40. Reduction of cell-cell adhesion is crucial for cancer cells departing from the primary tumor. AMP-activated protein kinase (AMPK) is well known for maintaining energy homeostasis. A recent study revealed that PI3K and HER2 activation transcriptionally downregulated AMPKα1 expression via ΔNp63α and highlighted that transcriptional control was another layer of AMPK regulation, which suggested the pivotal role of AMPKα1 in cell-cell adhesion and cancer metastasis (Fig. 2) 41. S100 calcium binding protein A14 (S100A14) promoted cancer metastasis by upregulating the expression and secretion of CCL2 and CXCL5 via RAGE/NF-κB pathway (Fig. 2) 42. These findings provided new therapeutic strategies for inhibiting breast cancer metastasis.

Long noncoding RNA (lncRNA) plays an important role in regulating TNBC metastasis. Antisense strand of nicotinamide phosphoribosyltransferase (NAMPT-AS) was an oncogenic lncRNA that epigenetically activated NAMPT to promote breast cancer metastasis (Fig. 2) 43. LncRNA LINC00665, encoding a micropeptide CIP2A-BP (CIP2A binding peptide), significantly reduced lung metastasis of breast cancer (Fig. 2) 44. LncRNA LINC00908, encoding a polypeptide ASRPS (a small regulatory peptide of STAT3), inhibits angiogenesis and metastasis of TNBC (Fig. 2) 45.

In addition, Nie et al. raised new precautions in dexamethasone therapy for breast cancer patients. Glucocorticoids (GCs) could activate TEA domain transcription factor 4 (TEAD4) in the Hippo signaling pathway for cancer metastasis and chemo-resistance by enhancing the interaction of the glucocorticoid receptor (GR) with TEAD4 to form a transcriptional complex (Fig. 2) 46.

## Drug resistance

Drug resistance, both intrinsic and acquired, remains the main obstacle for effective anticancer therapies.

In terms of chemotherapy, cancer cells can develop drug resistance by activating DNA damage repair signaling pathways 47. Following DNA damage, poly ADP-ribose polymerase 1 (PARP1) recruits MORC2 (MORC family CW-type zinc finger 2) to DNA damage sites and catalyzes MORC2 PARylation, which enhances its chromatin remodeling activities, thereby facilitating efficient DNA repair. In turn, MORC2 stabilizes PARP1 through a crosstalk between N-acetyltransferase (NAT10)-mediated acetylation and CHFR-mediated ubiquitination 48. DNA-damaging chemotherapeutic agents stimulate MORC2 acetylation in a NAT10 dependent manner, then acetylated MORC2 activates G2 DNA damage checkpoint, enhancing cell survival following DNA damage 49. Therefore, depleting MORC2 or inhibiting NAT10 sensitizes cancer cells to chemotherapy by abrogating DNA damage repair. Moreover, Rac 1 enhances the activity of non-oxidative pentose phosphate pathway via activating aldolase A and ERK signaling pathway, thereby inhibiting the nucleotide metabolism and DNA damage caused by chemotherapy in breast cancer 50. Additionally, Zhang et al revealed that a feed-forward circuit between serglycin (SRGN) and YES-associated protein (YAP) induced HDAC2 expression, promoting chemoresistance to 5-FU 51. Dong et al. found glutathione S-transferases P1 (GSTP1) contributed to adriamycin resistance 52. In TNBC, synaptotagmin-like 4 (SYTL4) contributes to taxane resistance via attenuating the stability of microtubule network and increasing the growth rate of microtubule 53 The antisense intronic lncRNA (ai-lncRNA) EGOT (eosinophil granule ontogeny transcript) sensitizes breast cancer cells to paclitaxel by enhancing autophagy^54^. MEF2-interacting transcriptional repressor (MITR), as a truncated isoform of HDAC9, induces resistance of paclitaxel via increasing interleukin-11 (IL11) expression and subsequent activating JAK/STAT3 signaling pathway 55.

In terms of endocrine therapy, antiestrogens stabilize MORC2 in a GPER1 (G protein coupled estrogen receptor 1) dependent manner and decrease MORC2 enhanced cellular sensitivity to tamoxifen and fulvestrant 56. In estrogen receptor (ER) positive breast cancer, lncRNA BDNF-AS, driven by a MEF2A regulated enhancer, induces endocrine resistance of breast cancer by activating the RNH1 (ribonuclease inhibitor 1)/TRIM21/mTOR (mechanistic target of rapamycin) cascade 57. This is a novel mechanism other than the canonical PI3K (phosphatidylinositol-3-kinase)/AKT/mTOR axis. LncRNA DILA1 contributes to tamoxifen resistance in breast cancer via interacting with Cyclin D1 and blocking its phosphorylation and degradation 58. Ajuba recruits DBC1 and CBP/p300, forming a ternary complex, to increase transcriptional activity of ERα and to potentiate the ERα target gene expression, thereby contributing to tamoxifen resistance 59. MiR-575 enhances ERα activity and tamoxifen resistance via targeting cyclin dependent kinase inhibitor 1B (CDKN1B) and BRCA1 60. Moreover, inactivation of neddylation with MLN4924 transcriptionally inhibits ERα and significantly improves the fulvestrant sensitivity 61. However, in ER negative breast cancer, targeting ubiquitin carboxyl terminal hydrolase-L1 (UCH-L1) upregulates ERα expression via enhancing ubiquitination and degradation of EGFR, and subsequently increases the sensitivity of tamoxifen and fulvestrant 62.

In terms of targeted therapy, lncRNA TINCR induces trastuzumab resistance by regulating miR-125b/HER-2 (human epidermal growth factor receptor 2) pathways 63. LncRNA TROJAN promotes ER+ breast cancer resistance to palbociclib, a cyclin-dependent kinase 4 and 6 (CDK4/6) inhibitor, through TROJAN-NKRF-CDK2 axis 64. PARP inhibitor olaparib is effective in treating breast cancer patients with BRCA1 mutations. All-trans retinoic acid (ATRA) sensitizes BRCA1-proficient breast cancer to PARP inhibition by inhibiting Pin1 and destabilizing BRCA1, which could extend the use of PARP inhibitors 65.

**Figure 3:**
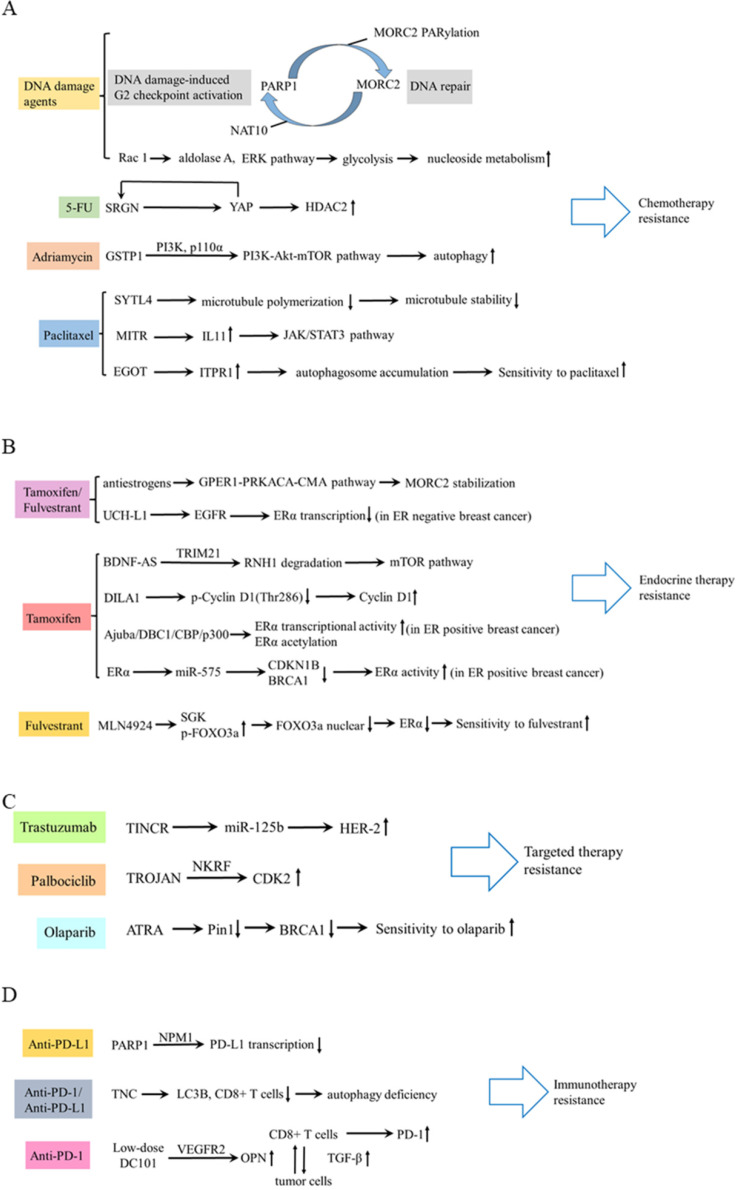
** Schematic diagram of drug resistance.** (A) The mechanism of drug resistance in chemotherapy, including DNA damage agents, 5-FU, Adriamycin, and paclitaxel. (B) Drug resistance mechanism of tamoxifen/fulvestrant, tamoxifen and fulvestrant. (C) Drug resistance mechanism of trastuzumab, palbociclib and olaparib. (D) Drug resistance mechanism of immunotherapy.

In regard to immunotherapy, breast cancers have poor response to immunotherapy because of poor T-cell infiltration and heightened immunosuppression within TME 66. A low-dose vascular endothelial growth factor receptor 2 (VEGFR2) blockade can increase immune cell infiltration and upregulate programmed cell death protein-1 (PD-1) expression on immune cells, thus sensitizing breast cancer to a PD-1 blockade 67. PARP1 suppresses PD-L1 expression via interaction with nucleophosmin (NPM1), which abolishes the binding of NPM1 at the *PD-L1* gene promoter in TNBC. Olaparib elevates PD-L1 expression and leads to better anti-cancer efficacy in combination with the anti-PD-L1 antibody 68. In addition, as a strong candidate for autophagy deficiency mediated immunosuppression, Tenascin-C (TNC) is overexpressed and is inversely correlated with LC3B expression and CD8+ T cells in TNBC patients. Inhibition of TNC in autophagy impaired TNBC cells sensitizes T cell mediated tumor killing and boosts anti-tumor effects of anti-PD-1/PD-L1 therapy 69. These findings provided new approaches to potentially combine immunotherapy with molecular-targeting agents. These drug resistance mechanisms aforementioned are summarized in Table 1 and Figure 3.

## Tumor biomarkers

The prognosis of breast cancer mainly depends on early detection and intervention. Recently, accumulated evidence indicates that circulating tumor DNA (ctDNA) could be a sensitive and specific biomarker for monitoring breast cancer progression and predicting relapse in early-stage 70. A study showed the positive detection rate of ctDNA in early-stage breast cancer could reach 74.2%. Moreover, positive ctDNA after surgery indicated potential recurrence and remote metastasis 71. Serum LRP6 ectodomain (LRP6N) could be another promising metastatic diagnosis marker for its binding to CXCR4 and competitively preventing SDF-1/CXCR4-induced lung metastasis 72.

Because of the highly heterogeneity of TNBC, appropriate classification will provide effective prediction for outcomes. TNBC were classified into three heterogeneous clusters based on the microenvironment phenotypes: cluster 1, the “immune-desert” cluster, with low microenvironment cells infiltration; cluster 2, the “innate immune-inactivated” cluster, with resting innate immune cells and nonimmune stromal cells infiltration; and cluster 3, the “immune-inflamed” cluster, with abundant adaptive and innate immune cells infiltration. Cluster 1 and 2 were both called “cold tumors” while cluster 3 was a “hot tumor”. Immune checkpoint inhibitors might be effective for “immune-inflamed” clusters, indicating that these distinct phenotypes were potential biomarkers for predicting therapeutic efficacy. The transformation of “cold tumors” into “hot tumors” should also be considered when dealing with “immune-desert” and “innate immune-inactivated” clusters 73.

The molecular typing of breast cancer has been revealed deeply by novel detecting techniques. A study divided Chinese TNBC into four subtypes based on multi-omics data: luminal androgen receptor, immunomodulatory, basal-like immune-suppressed, and mesenchymal-like subtypes, which could serve as a reference in order to further advance the understanding and precision treatment of TNBC 74. Further, the same group conducted a phase Ib/II trial, demonstrating the clinical benefit of molecular subtyping-based and targeted sequencing-based therapy for refractory metastatic TNBC. Specifically, nab-paclitaxel in combination with anti-PD-1 therapy or with anti-VEGFR therapy showed favorable outcomes on IM (immunomodulatory) subtype and *BRCA1/2* wild type-BLIS (basal-like immune-suppressed) subtypes, respectively 75. Moreover, based on distinct metabolic dysregulation, TNBC was classified into three subtypes via multi-omics database analysis. MPS1 (heterogeneous metabolic-pathway-based subtype 1) is the lipogenic subtype with upregulated lipid metabolism. MPS2 is the glycolytic subtype with upregulated carbohydrate and nucleotide metabolism. MPS3 is the mixed subtype with partial pathway dysregulation. Different subtypes respond to metabolic inhibitors with distinct sensitivity which enables the development of personalized cancer therapy by targeting unique metabolic profiles 76.

## Novel therapeutic targets and new drugs

With the increasing popularity of precision therapy for breast cancer, it is urgent to develop new therapeutic targets and drugs.

Recent studies showed the effects of FTO^77^, protein arginine methyltransferase 1 (PRMT1) 78, coactivator associated arginine methyltransferase 1 (CARM1)/PRMT4 79, Otubain-2 (OTUB2) 80, RING finger protein 144A (RNF144A) 81, plant homeodomain finger protein 20-like protein 1 (PHF20L1) 82, heat shock transcription factor 1 (HSF1) 83, epithelial cell transforming sequence 2 (ECT2) 84, pescadillo homolog 1 (PES1) 85, PHACTR2-AS1 (PAS1) 86, breast cancer-related transcript 1 (BCRT1) 87, and circCDYL 88 on progression of breast cancer and they provided novel therapeutic targets. TNBC is especially hard to treat due to the lack of targets. A recent study demonstrated NOTCH1-ATR-CHK1 cascade and cisplatin displayed good synergy in inhibiting TNBC by targeting cell cycle checkpoint, DNA damage, and EMT 89. In addition, TNBC progression was also promoted by TROJAN 90, circSEPT9 91, and moesin (MSN) 92, which could be potent options for this fatal disease. These potential novel therapeutic targets are summarized in Table 2.

As we know, tumor metabolism is important in cancer progression. Nevertheless, the lack of effective and selective anti-metabolism drugs hinders clinical application. Chen et al reported a small molecule ZY-444 bound to pyruvate carboxylase, which was a key anaplerotic enzyme of the tricarboxylic acid cycle in promoting cancer growth and metastasis through Wnt/β-catenin/Snail signaling pathways. ZY-444 suppressed breast cancer cell proliferation with low cytotoxicity in normal cells by inactivating catalytic activity of pyruvate carboxylase 93. Flubendazole, a broad-spectrum anthelmintic drug belonging to benzimidazole group, has been repurposed as a promising anti-cancer agent 94. Zhen et al revealed flubendazole induced autophagic cell death by targeting Eva-1 homolog A (EVA1A) and suppressed TNBC proliferation and migration 95. Moreover, resistomycin, QN-1 and emodin attenuates TNBC progression via binding to Pellino-1 to promote FBXO11-mediated SNAIL/SLUG degradation 96, down-regulating c-MYC transcription 97 and reducing VEGFA transcription 98, respectively. Accordingly, these studies provided alternative strategies for treating TNBC.

## Conclusion

In summary, Chinese investigators have made significant progress in all areas of breast cancer research over the last two years, particularly in the field of BCSCs. A number of new BCSC membrane biomarkers, including PROCR, NOTCH4, SGCE, TSPAN8, and IL1R2, were identified. They may be used for diagnosis and potential therapeutic targets. In addition, stemness of BCSCs could be regulated by tumor microenvironment, such as TAM, CAF, and signaling molecules. Due to the increased patient numbers, limited treatment options, and unsatisfactory treatment efficacy in China, investigators focused on the exploration of drug resistance and metastasis mechanisms and made great efforts to identify new tumor biomarkers and therapeutic targets in order to improve the diagnosis and treatment of breast cancer. However, there is still a lack of milestone discovery in terms of breast cancer cell origin, pathological typing, and targeted metastatic therapy. Thus, multi-party cooperation is strongly recommended to make a breakthrough in basic research and clinical translation.

## Figures and Tables

**Table 1:** Drug resistance mechanisms of breast cancer

Drug resistance	Drugs	Mechanism	Reference
Chemotherapy	DNA damage agents	A feedback loop between MORC2 and PARP1 facilitates efficient DNA repair	48
		NAT10-mediated MORC2 acetylation contributes to DNA damage-induced G2 checkpoint activation	49
		Rac1 promotes the glycolysis, especially non-oxidative pentose phosphate pathway and nucleoside metabolism	50
	5-FU	A feed-forward circuit between SRGN and YAP induces HDAC2 expression to maintain stemness and chemoresistance	51
	adriamycin	GSTP1 promotes autophagy by interacting with PI3K, p110α, and then preventing PI3K-Akt-mTOR pathway signaling	52
	paclitaxel	SYTL4 decreases microtubule stability via inhibiting microtubule polymerization	53
		EGOT enhances autophagosome accumulation via the upregulation of ITPR1 expression in cis and in trans	54
		MITR increases IL11 expression and activation of downstream JAK/STAT3 signaling pathway	55
Endocrine therapy	tamoxifen/fulvestrant	Estrogen receptor antagonists stabilize MORC2 via the GPER1-PRKACA-CMA pathway	56
		UCH-L1 contributes to loss or reduction of ERα by the deubiquitinase-mediated stability of EGFR	62
	tamoxifen	BDNF-AS promotes RNH1 degradation via TRIM21-mediated ubiquitination and sustains the activation of mTOR signaling	57
		DILA1 blocks phosphorylation and degradation of Cyclin D1	58
		Ajuba*/*DBC1*/*CBP*/*p300 ternary complex co-activates ERα transcriptional activity and enhances ERα acetylation	59
		miR-575 enhances ERα activity by targeting CDKN1B and BRCA1	60
	fulvestrant	Inactivation of neddylation with MLN4924 inhibits ERα via delaying SGK degradation and inducing FOXO3a nuclear export	61
Targeted therapy	trastuzumab	TINCR promotes HER-2 expression by sponging miR-125b and promotes EMT by targeting Snail-1	63
	palbociclib	TROJAN binds to NKRF and inhibits its interaction with RELA, upregulating the expression of CDK2	64
	olaparib	ATRA sensitizes BRCA1-proficient breast cancer to PARP inhibition by inhibiting Pin1 and destabilizing BRCA1	65
Immunotherapy	anti-PD-1	Low-dose VEGFR2 blockade sensitizes tumors to anti-PD-1 therapy via upregulation of PD-1 on immune cells through stimulating the secretion of OPN and TGF-β	67
	anti-PD-L1	PARP1 suppresses PD-L1 transcription through interacting with NPM1 and abolishing the binding of NPM1 at the PD-L1 promoter	68
	anti-PD-1/anti-PD-L1	TNC contributes to autophagy deficiencymediated immunosuppression via suppressing LC3B and CD8+ T cells	69

PRKACA protein kinase cAMP-activated catalytic subunit alpha, CMA chaperone-mediated autophagy, SGK serum and glucocorticoid-induced protein kinase, FOXO3a forkhead box O3a, OPN osteopontin.

**Table 2:** Potential novel therapeutic targets of breast cancer

Target	Function	Mechanism	Reference
PES1	Promotes breast cancer growth	Forms a complex with TERT and the TR, regulating telomerase activity, telomere length maintenance, and senescence	85
MSN	Stimulates TNBC cells proliferation and invasion	Phosphorylated MSN interacts with the nucleoprotein NONO and promotes the nuclear localization of PKC interacting with MSN, which leads to the phosphorylation of CREB and the up-regulation of downstream gene expression	92
PHF20L1	Maintains the proliferative state of breast cancer cells	recognizes H3K27me2 and collaborates with PRC2 and the NuRD complex in regulating H3K27 modifications to suppress a series of tumor suppressors	82
TROJAN	Promotes TNBC cells proliferation and invasion	Increases ZMYND8 degradation and epigenetically upregulates metastasis-related genes	90
PAS1	Inhibits breast cancer cells proliferation and metastasis	Binds to rDNA genes and recruits histone methyltransferase SUV39H1, triggering H3K9 methylation of these genes, resulting in the suppression of ribosome synthesis	86
BCRT1	Promotes breast cancer cells proliferation and mobility	competitively binding with miR-1303 to protect PTBP3 from degradation; promotes M2 polarization; facilitates hypoxia-induced EMT	87
circCDYL	Promotes breast cancer cells proliferation	promotes breast cancer malignant progression via the miR-1275-ATG7/ULK1-autophagic axis	88
circSEPT9	Promotes TNBC cells proliferation, migration, invasion and inhibits TNBC cells apoptosis and autophagy	E2F1 and EIF4A3 mediated circSEPT9 regulates the expression of LIF via sponging miR-637 and activates LIF/Stat3 signaling pathway	91
OTUB2	Promotes breast cancer stemness and metastasis	EGF and KRAS mutation induce OTUB2 poly-SUMOylation, thereby deubiquitinates and activates YAP/TAZ	80
NOTCH1	Induces the TNBC formation	Promotes the EMT and regulates the cell cycle through activation of ATR-CHK1 signalling pathway	89
PRMT1	Promotes breast cancer cells proliferation	PRMT1-dependent methylation of C/EBPα promotes the expression of cyclin D1 by blocking the interaction between C/EBPα and HDAC3	78
CARM1	Promotes ERα-positive breast cancer cells proliferation	Transcriptional activates cognate estrogen-induced genes and methylates a large cohort of proteins	79
FTO	Promotes breast cancer cells proliferation, colony formation and metastasis	Demethylates N6-methyladenosine in the 3'UTR of BNIP3 and causes its degradation	77
RNF114A	Suppresses breast cancer cells proliferation, colony formation, migration, and invasion	Interacts with and targets HSPA2 for ubiquitination and degradation	81
HSF1	Promotes breast cancer cells proliferation, migration, and invasion	PIM2-mediated HSF1 phosphorylation at Thr120 promotes proteostasis and carboplatin-induced autophagy, and enhances PD-L1 expression	83
ECT2	promotes breast cancer cells survival	Forms a positive feedback loop with USP7 to promote stabilization of each other, ultimately sustains the expression of MDM2	84

TERT telomerase reverse transcriptase, TR telomerase RNA, PKC protein kinase C, CREB cAMP response element-binding protein, PRC2 polycomb repressive complex 2, NuRD Mi-2/nucleosome remodeling and deacetylase, ZMYND8 zinc finger MYND-type containing 8, rDNA ribosome DNA, LIF leukemia inhibitory factor, TAZ WW domain-containing transcription factor, C/EBPα CCAAT/enhancer binding protein α, HSPA2 heat-shock protein family A member 2,PIM proviral integration site for moloney murine leukemia virus.
